# Purinergic Signaling During Hyperglycemia in Vascular Smooth Muscle Cells

**DOI:** 10.3389/fendo.2020.00329

**Published:** 2020-05-22

**Authors:** Miguel Martin-Aragon Baudel, Ricardo Espinosa-Tanguma, Madeline Nieves-Cintron, Manuel F. Navedo

**Affiliations:** ^1^Department of Pharmacology, University of California, Davis, Davis, CA, United States; ^2^Departamento de Fisiologia y Biofisca, Universidad Autónoma San Luis Potosí, San Luis Potosí, Mexico

**Keywords:** purinergic receptors, diabetes, ion channels, myogenic tone, vascular reactivity, P2Y_11_

## Abstract

The activation of purinergic receptors by nucleotides and/or nucleosides plays an important role in the control of vascular function, including modulation of vascular smooth muscle excitability, and vascular reactivity. Accordingly, purinergic receptor actions, acting as either ion channels (P2X) or G protein-coupled receptors (GCPRs) (P1, P2Y), target diverse downstream effectors, and substrates to regulate vascular smooth muscle function and vascular reactivity. Both vasorelaxant and vasoconstrictive effects have been shown to be mediated by different purinergic receptors in a vascular bed- and species-specific manner. Purinergic signaling has been shown to play a key role in altering vascular smooth muscle excitability and vascular reactivity following acute and short-term elevations in extracellular glucose (e.g., hyperglycemia). Moreover, there is evidence that vascular smooth muscle excitability and vascular reactivity is severely impaired during diabetes and that this is mediated, at least in part, by activation of purinergic receptors. Thus, purinergic receptors present themselves as important candidates mediating vascular reactivity in hyperglycemia, with potentially important clinical and therapeutic potential. In this review, we provide a narrative summarizing our current understanding of the expression, function, and signaling of purinergic receptors specifically in vascular smooth muscle cells and discuss their role in vascular complications following hyperglycemia and diabetes.

## Introduction

Nucleotides can act as extracellular signaling molecules engaging plasma membrane bound purinergic receptors in different tissues (1). In endothelial cells, these receptors mediate nitric oxide production and relaxation (2). In vascular smooth muscle cells, purinergic receptors primarily mediate vasoconstrictive actions (3–7), although there are some examples in which they can also mediate vasorelaxation (8–10). This dual short-term control by purinergic receptors contributes to the regulation of vascular reactivity and myogenic tone. Purinergic receptors are also involved in the long-term development of trophic events by participating in cell proliferation, differentiation, migration, and death, all of which are associated with development of vascular diseases (11). Nucleotides are released from endothelial cells due to shear stress, hypoxia, and low pH, which together with neural release of adenosine triphosphate (ATP) and uridine triphosphate (UTP) acting on smooth muscle cells, contribute to the regulation of blood flow in the vascular system. Besides the granular ATP secretion by platelets and nerve terminals through vesicular exocytosis, non-vesicular release of nucleotides occurs in virtually all cells (12). Such release occurs upon agonist, chemical, or mechanical stimulation, appearing to involve a variety of anionic pore-forming membrane proteins, such as pannexins, connexins, P2X_7_ receptors, or ATP-binding cassette transporters (13). Extracellular breakdown of ATP and UTP is also necessary for purinergic signaling in the vasculature and is mediated by ectonucleotidases, including ectonucleoside triphosphate diphosphohydrolase (E-NTPDases), ectonucleotide pyrophosphatase/phosphodiesterase (E-NPPS), alkaline phosphatase, and ecto-5′-nucleotidose (14). The combined action of purinergic receptors together with purinergic transporters and converting enzymes create a complex signaling network that has been argued to play a central role in different pathological conditions, including diabetic hyperglycemia (15).

Vascular function gradually decline with age manifesting as biochemical and structural changes in blood vessel function that compromises vascular health (16). Diabetes is a complex chronic metabolic/cardiovascular disorder with multiple pathophysiological abnormalities and a recognized cause of accelerated vascular aging (17). Elevated blood glucose levels (e.g., hyperglycemia) is a defining characteristic of diabetes whether produced by insulin-deficiency (type 1 diabetes) or insulin-resistance (type 2 diabetes) (18, 19). Both hyperglycemia and diabetes promote vascular complications that increase the risk of suffering from hypertension, stroke, coronary disease, and organ failure (20, 21). Hyperglycemia and diabetes are also associated with decreased cognitive function, retinopathy, and nephropathy (22). Hyperglycemia-induced vascular complications are due in part to altered vascular reactivity (23). In addition to endothelial dysfunction, which results in impaired vascular regulation, endothelium-independent mechanisms, including altered vascular smooth muscle cell excitability, are emerging as critical in the development, and progression of vascular complications in diabetic hyperglycemia (24–27). It has to be noted that both reduced and enhanced vasoconstriction have been described in both human and animal models of diabetes (28–32). Intriguingly, purinergic signaling has been observed to be altered in endothelial and vascular smooth muscle from both experimental animal models and humans with type-2 diabetes (33–35). Due to the involvement of purinergic receptors in regulating vascular tone, they may represent potential targets for the treatment of vascular complications during diabetic hyperglycemia. The contributions of purinergic signaling to endothelial cell function in health and disease has been extensively examined in recent studies (1, 2, 36–38). Here, we focus on how purinergic receptors regulate vascular smooth muscle function in health, in response to hyperglycemia, and during diabetes.

Functional expression of ion channels, which may be modulated by purinergic signaling (39–41), regulate vascular smooth muscle excitability and therefore vascular reactivity and myogenic tone (42). Vascular reactivity is the response of blood vessels to constrict or dilate in response to a given stimulus while myogenic tone refers to a sustained state of smooth muscle contraction. The expression and function of different types of K^+^ channels as well as the L-type Ca^2+^ channel Ca_V_1.2 are essential for modulation of vascular reactivity and myogenic tone (42), and their functional expression is altered in response to elevated glucose and diabetes (25, 27, 42, 43). Changes in vascular smooth muscle ion channels' functional expression during hyperglycemia and diabetes can be underlined by activation of purinergic signaling. Accordingly, recent exciting findings have revealed a novel mechanism involving purinergic signaling in the regulation of L-type Ca^2+^ channels in vascular smooth muscle upon acute hyperglycemia exposure and during diabetes. This mechanism may contribute to modulate vascular smooth muscle excitation-contraction and excitation-transcription coupling. The findings revealed an elegant signaling complex that is engaged in response to hyperglycemia and diabetes and may have important clinical and therapeutic implications. In this review, we summarize current knowledge about the expression and function of purinergic receptors in vascular smooth muscle. Our main goals are to discuss prior literature and exciting findings describing the effect of hyperglycemia and diabetes on purinergic signaling and how it alters vascular smooth muscle function and vascular reactivity during this pathological condition/stimulus.

## Expression and Function of Purinergic Receptors in Vascular Smooth Muscle

It is now well-established that purinergic signaling plays a pivotal role in the control of vascular smooth muscle function and corresponding regulation of myogenic tone and vascular reactivity (44, 45). The release of ATP, UTP, and/or its breakdown products from either endothelial cells, epithelial cells, platelets or sympathetic, and sensory-motor nerves, can induce either vasoconstrictor and vasodilatory effects through the activation of purinergic receptors in vascular smooth muscle cell (1, 46). Indeed, the endothelium itself could release as much as 300 nM ATP to the extracellular milieu (47), which may activate purinergic signaling in adjacent vascular smooth muscle cells. In addition, autocrine nucleotide release from vascular smooth muscle cells during hyperglycemia has been associated with changes in Ca^2+^ signaling, activation of transcription factors, and modulation of cell excitability, which seems to be mediated via engagement of one or more purinergic receptors (48, 49). Furthermore, in a rat model of streptozotocin (STZ)-induced diabetes (50), zebrafish (51), and retinal cultures (52) exposed to elevated extracellular glucose, and both type 1 (53) and type 2 diabetic patients (53–55), purine blood and/or extracellular levels have been shown to be elevated, which could be responsible for increased purinergic receptor signaling.

The first evidence for purines having a physiological effects in the cardiovascular system was reported more than 90 years ago (56). Two different families of purinergic receptors were subsequently identified and classified according to their primary agonist: (1) adenosine conforming the P1 family and (2) ATP/UTP corresponding to the P2 family (57). Four P1 GPCR subtypes (A_1_, A_2A_, A_2B_, and A_3_), 7 P2X ion channel receptor subtypes (P2X_1−7_), and 8 P2Y GPCR subtypes (P2Y_1_, P2Y_2_, P2Y_4_, P2Y_6_, P2Y_11_, P2Y_12_, P2Y_13_, and P2Y_14_) are recognized (58, 59). Different purinergic receptors have been identified in vascular smooth muscle cells of different vascular beds and species, including P2X_1_, P2X_2_, P2X_4_, P2X_5_, P2Y_1_, P2Y_2_, P2Y_4_, P2Y_6_, P2Y_11_, P2Y_12_, P1A_1_, P1A_2_, and P1A_3_ (1). These purinergic receptors have been shown to mediate both vasoconstrictor and vasodilatory effect in a species- and vascular bed-dependent manner, as well as having a role in vascular smooth muscle cells differentiation and proliferation (4, 48, 49, 60). In the following section, we will describe each family and receptor subtype and their involvement in the regulation of the vascular system highlighting their role in hyperglycemia and diabetes. Table 1 summarizes the expression, physiological agonist, physiological effect and involvement in pathophysiology of different purinergic receptors in smooth muscle cells from different vascular beds.

**Table 1:** Summary of the different purinergic receptors expressed in smooth muscle cells discussed in this review.

**Purinergic receptor**	**Arterial bed expression (species)**	**Agonist**	**Smooth muscle cell effect**	**Ion channel/GPCR type**	**Involvement in vascular disease**	**References**
P2X_1_	Mesenteric (mouse, rat), aorta, renal (rat)	ATP, Up_4_A, α,β-meATP	Vasoconstriction	Ion channel	Obesity, diabetes	(3, 4, 61, 62)
P2X_1_/P2X_4_	Cerebral mesenteric, femoral, pulmonary, coronary and renal (rat), omental (human)	ATP	Vasoconstriction	Ion channel		(63–66)
P2X_5_	Mesenteric (rat)		No functional role reported	Ion channel		(67)
P2Y_1_	Chorionic and mammary (human), intrapulmonary (rat)	ATP, ADP	Vasodilation	G_q_		(10, 68–70)
P2Y_2_	Coronary (mouse, human), pulmonary (rat)	ATP, UTP	Vasoconstriction	G_q_		(6, 71, 72)
P2Y_4_	Cerebral, mesenteric (rat)	ATP, UTP	Vasoconstriction	G_q_	Diabetes	(60, 73, 74)
P2Y_6_	Aorta, mesenteric, basilar, coronary (mouse)	UDP, UDP-glucose	Vasoconstriction	G_q_	Hypertension, hyperglycemia	(3, 6, 48, 75–77)
P2Y_11_	Pulmonary (rat), adipose (human), cerebral and mesenteric (mouse)	ATP, UTP	Vasoconstriction	G_q/11_/G_s_	Hyperglycemia	(49, 78, 79)
P2Y_12_	Mammary, pericardial fat arteries (human), aorta (mouse)	ADP	Vasoconstriction	G_i/o_	Atherosclerosis	(80–83)
P1A_1_	Aorta (mouse)	Adenosine	Vasoconstriction	G_i_		(84, 85)
P1A_2A_	Coronary (mouse)	Adenosine, Up_4_A	Vasodilation	G_s_		(9, 86, 87)
P1A_2B_	Chorionic (human)	Adenosine	Vasoconstriction, Vasodilation	G_s_		(86, 88)
P1A_3_	Aorta (mouse)	Adenosine	No functional role reported	G_i_		(89)

### P2X Receptors in Vascular Smooth Muscle Cells

P2X receptors are cation permeable ligand-gated ion channels (90). Their activation by ATP leads to a rapid response involving Ca^2+^ and Na^2+^ entry directly through the P2X channel pore (91). The subsequent membrane depolarization of vascular smooth muscle due to activation of P2X channels contribute to calcium influx via voltage-gated L-type Ca^2+^ channels. The source of ATP for activation of P2X channels may come from sympathetic nerves in the adventitia as a co-transmitter with noradrenaline and neuropeptide Y, but also from contracting smooth muscle or damaged cells to induce vasoconstriction (1, 13, 92, 93)

The contractile actions of ATP released from perivascular sympathetic nerves in smooth muscle cells was confirmed to involve principally homomeric P2X_1_ receptors (3). In this study, the involvement of P2X_1_ receptors was corroborated using P2X_1_ knockout mice. P2X_1_ receptors appear in close proximity to sympathetic nerve varicosities where they form clusters that seem to be associated with lipid rafts (94, 95). Depolarizations mediated by neural release of ATP engaging P2X_1_ receptors are known as excitatory junction potentials, which can summate to produce vasoconstriction involving L-type Ca^2+^ channel activation (96–98). This process is vessel-dependent, as rat mesenteric artery vasoconstriction is entirely mediated via the receptor pore (97). Noradrenaline, which is co-released with ATP by sympathetic nerves, induces longer depolarizations, and contractions as it involves α-adrenoceptor coupling to G_q_ proteins. This elicits Ca^2+^ release from internal stores via inositol 1,4,5-trisphosphate (IP_3_), and this combined rise in cytoplasmic Ca^2+^, together with Ca^2+^ sensitization induces contraction (99). The vasoconstrictive contribution of ATP and noradrenaline is also vessel-dependent, with ATP mediating 10% of the peak response in rat tail artery (100), 20–60% in rabbit central ear artery (101) and 100% in rabbit mesenteric artery (102). Thus, sympathetic nerve-mediated vasoconstriction and the relative contribution of noradrenaline and ATP is influenced by many factors. In rat intrapulmonary arteries, ATP released by non-adrenergic non-cholinergic nerves (NANC) can also elicit excitatory junction potential (103). This is in contrast to most blood vessels in which ATP released from NANC nerves induces endothelium-dependent vasorelaxation (104, 105).

Uridine adenosine tetraphosphate (Up_4_A) is an endothelium-derived vasoconstricting factor that when released by different stimuli engages P2X_1_ receptors to promote contraction of rat aortic vascular smooth muscle cells (4), and perhaps cells in other vascular beds. This contraction was attenuated by L-type Ca^2+^ channel antagonists and Rho-kinase inhibitors (4), suggesting a crosstalk between P2X_1_ receptor, L-type Ca^2+^ channels, and Rho-kinase signaling that regulates vascular reactivity.

Other P2X receptors have been recently identified in vascular smooth muscle cells. For example, heteromeric P2X_1_/P2X_4_ receptors have been shown to mediate vasoconstriction of rat cerebral arteries (63). Heteromeric P2X_1_/P2X_4_ receptors are also expressed in human omental arteries (64) and rat mesenteric, femoral, pulmonary, coronary, and renal arteries (65, 66, 106). The functional role of these purinergic receptors in these vascular beds as well as their role in health and disease, however, remains to be elucidated. P2X_5_ receptors are also expressed in rat small mesenteric small arteries with no functional role reported to date (67).

### P2Y Receptors in Vascular Smooth Muscle Cells

P2Y receptors are GPCRs with ligand selectivity (107). ATP is the primary physiological P2Y_11_ agonist. P2Y_2_ and P2Y_4_ are activated by ATP but also by UTP. Adenosine diphosphate (ADP) activates P2Y_1_, P2Y_12_, and P2Y_13_. Finally, P2Y_6_ and P2Y_14_ are activated by uridine diphosphate (UDP) and UDP-glucose, respectively. In addition, the G protein subtype of each receptor defines the specificity of the intracellular signal elicited (107). For example, P2Y_1_, P2Y_2_, P2Y_4_, and P2Y_6_ couple primarily with G_q_, P2Y_12_, P2Y_13_, and P2Y_14_ couple with G_i/o_ and P2Y_11_ couple to both G_s_ and G_q/11_ in vascular smooth muscle cells (59, 78, 108). Given the number of different P2Y receptor subtypes, the variety of functional signaling cascades engaged, the biased agonism of GPCR activation and the relative lack of subtype-specific agonists and inhibitors, research into the (patho)physiological role of these receptors has been challenging and remains poorly understood.

P2Y_1_ receptors are mainly expressed in endothelial cells mediating vasodilation (109). Although P2Y_1_ expression has been detected in vascular smooth muscle cells from human mammary arteries (68) and in the rat intrapulmonary artery at low levels, they seem to play no role in the response to ATP in this tissues (10). In endothelium-denuded human chorionic arteries, P2Y_1_ expression has been shown to be higher (69) and to mediate vasoconstriction (70). Interestingly, this study demonstrates a micro-regionalized distribution of P2Y_1_ receptors into lipid rafts, which when disrupted, abrogated P2Y_1_ receptor-mediated vasoconstriction. Interestingly, differential expression of P2Y_1_ was observed along the human placental vascular tree, with a decline in receptor expression in the vascular smooth muscle layer as the tree approaches the capillary network (69). A concomitant reduction in agonist-mediated vasoconstriction and a shift in vascular response to vasodilation was observed as the size of the vessels decreased. Robust P2Y_1_ expression was also found in canine coronary vascular smooth muscle cells (109). In this study, P2Y_1_ activity was found to promote agonist-induced vasodilation of coronary arteries via an endothelium-dependent mechanism in *in vitro* and *in vivo* settings. However, these receptors did not seem to play a role in pressure-flow autoregulation, thus revealing distinct mechanisms by which P2Y_1_ can control vascular reactivity.

P2Y_2_ receptors are expressed in smooth muscle cells of coronary arteries in different species and their activation by ATP or UTP has been shown to promote vasoconstriction (6, 71). In small pulmonary veins, ATP-induced vasoconstriction was associated with the stimulation of P2Y_2_ receptors in vascular smooth muscle cells (72). This effect was linked to the activation of PLC-β and the generation of intracellular Ca^2+^ oscillations mediated by cyclic Ca^2+^ release events via IP_3_ receptor activation. In vascular smooth muscle cells, P2Y_2_ receptors also have trophic roles, stimulating DNA synthesis, proliferation, and migration of human and rat aortic vascular smooth muscle cells, which are key events in vascular remodeling (1).

P2Y_4_, which are selectively activated by pyrimidines, are present in smooth muscle cells of cerebral arteries where their activation leads to vasoconstriction (73). The mechanism by which P2Y_4_ are activated and mediate vasoconstriction in intraparenchymal cerebral arterioles is proposed to be mechanically linked instead of through the release of endogenous nucleotides, and likely involves activation of TRP channels, inhibition of K^+^ channels, or direct activation of L-type Ca^2+^ channels (60). P2Y_4_ receptors have also been shown to mediate proliferation of rat aortic smooth muscle cells (110).

Different studies in rodents have highlighted the role of P2Y_6_ receptor in vascular smooth muscle cells as mediator of contraction in aorta, mesenteric, and basilar arteries (3, 75, 76). In mouse large diameter segments of the coronary artery, P2Y_6_ activation promotes contraction of vascular smooth muscle in response to UDP, whereas in smaller diameter segments, its activation causes vasodilation via an endothelium-dependent mechanism (6). P2Y_6_ is the most expressed P2Y receptor in resistance arteries and contributes to the maintenance of the myogenic tone through an autocrine/paracrine activation loop (7). Interestingly this action is independent of intracellular Ca^2+^ increase through the G_q_/PLCβ/IP_3_ pathway and is proposed to involve phosphorylation of mitogen-activated protein kinases P38/P42–44/c-Jun N-terminal and the Rho-kinase Ca^2+^ sensitizing pathway (111). These mitogen-activated protein kinases are activated by different external stressors such as heat, UV irradiation, osmotic shock, or cell stretch (112–114). Therefore, P2Y_6_-mediated phosphorylation and activation of these kinases could represent a novel stress response mechanism. Kauffenstein et al., argued that cell stretching caused by a rise in intraluminal pressure induces the release of nucleotides that stimulate P2Y_6_ and promote smooth muscle cell contraction (autocrine/paracrine activation loop). However, other studies have suggested that the P2Y_6_ regulation of myogenic tone, at least in parenchymal arterioles, is not mediated by extracellular nucleotides, but rather by direct stretch-induced activation of the receptor (60). Yet, the mechanisms by which P2Y6 “sense” mechanical stretch remain to be elucidated. The importance of P2Y_6_ in regulating blood pressure has been recently highlighted by P2Y_6_ knockout mice that displayed attenuated angiotensin II (AngII) induced hypertension and vascular remodeling in mice (77). Intriguingly, this study also revealed heterodimer formation between angiotensin type 1 receptors (AT1R) and P2Y_6_ and an age-related increase of this heterodimerization (77), which could contribute to age-associated high blood pressure (115). P2Y_6_ activation by UDP has also been shown to act as a growth factor stimulating mitogenesis in vascular smooth muscle cells (116).

P2Y_11_ is primarily stimulated by ATP, although it can also be stimulated by ADP, but not pyrimidines (59, 78, 117). This receptor has the unique property of coupling to G_q/11_ and G_s_ proteins (78). Consequently, activation of P2Y_11_ can stimulate PLCβ/protein kinase C (PKC) and adenylyl cyclase (AC)/protein kinase A (PKA) signaling (78). However, its role in the vascular system has so far remained unclear. In cardiomyocytes, P2Y_11_ has been shown to mediate positive ionotropic effects via activation of PLC and cyclic adenosine monophosphate (cAMP) signaling, and its function seems to be impaired in a desmin-deficient mouse model of cardiomyopathy that produces congestive heart failure (118). Furthermore, a polymorphism (Ala-87-Thr) in P2Y_11_ has been associated to increased risk of acute myocardial infarction and C-reactive protein blood levels (119). In vascular smooth muscle cells from rat pulmonary arteries, a Ca^2+^-dependent chloride current was activated by ATP through a P2Y receptor that was suggested to resemble a P2Y_11_ receptor (79). In colonic smooth muscle, P2Y_11_ receptors are involved in both fast and slow relaxations through K_Ca_ channels conforming parasympathetic inhibition of the gut (120). However, its role in the vasculature has remained unclear. A recent study linked P2Y_11_ to glucose-mediated regulation of vascular smooth muscle excitability (further discussed below) (49).

The role of ADP-selective P2Y_12_ in platelet aggregation is well-recognized, being the target of the antithrombotic drug clopidogrel (121). Clopidogrel has also been shown to improve endothelial dysfunction in AngII-induced hypertensive rats by improving endothelial-mediated relaxation (122). However, this effect does not seem to be directly mediated by P2Y_12_ receptors expressed in the endothelium. P2Y_12_ has also been shown to be expressed in vascular smooth muscle cells and mediate contraction in human internal mammary arteries (80). In these experiments, clopidogrel did not reduced ADP-induced contraction, which was deemed to be due to the low penetration and high instability of this drug. A modified version of clopidogrel with increased half-life (AZD6140) has been shown to block P2Y_12_-mediated contractions in mice aorta and human internal mammary arteries and pericardial fat arteries (81). Recent evidence also suggests an increase in the expression of this receptor in vascular smooth muscle cells in atherosclerosis, and in playing a role in migration and potentiating atherogenesis (82, 83).

### P1A Receptors in Vascular Smooth Muscle Cells

P1 receptors are GPCRs that respond to adenosine upon hydrolysis of ATP (123). All P1 receptor subtypes expression (P1A_1_, P1A_2A_, P1A_2B_, and P1A_3_) have been described in smooth muscle cells where they mediate either vasoconstriction or vasorelaxation in a species- and vascular bed-specific form (45). P1 receptors A_2A_ and A_2B_ are coupled to AC through the activation of G_s_ proteins where they mediate relaxation, whereas A_1_ and A_3_ are coupled to G_i_ proteins and mediate vasoconstriction (124).

P1A_1_ receptors mediate aortic vascular smooth muscle cell contraction through the PLC pathway and its activation also reduces vascular smooth muscle relaxation mediated by P1A_2B_ and P1A_2A_ receptors (84). Activation of adenosine receptors leads to the metabolism of arachidonic acid via the PLC second messenger diacylglycerol (DAG). Studies utilizing P1A_1_ knockout (${\text{P}1\text{A}}_{1}^{-/-}$) mice demonstrated that arachidonic acid metabolites can activate PKCα and the ERK1/2 pathway in aortic vascular smooth muscle leading to vasoconstriction (5). Furthermore, PKCα activation via P1A_1_ receptors can lead to the inhibition of large-conductance Ca^2+^-activated K^+^ (BK_Ca_) channel activity (125), which can further contribute to contraction of vascular smooth muscle cells. P1A_1_-deficient mice have also been shown to present impaired autoregulation of the renal vascular resistance by removal of the P1A_1_-dependent vasoconstrictor tone (126), suggesting the involvement of the purinergic system in pressure-induced resistance changes. P1A_3_ receptor has been shown to mediate aortic vasoconstriction through an endothelium-dependent mechanism that is associated with reactive oxygen species (ROS) generation via Nox2 (89). However, the role that vascular smooth muscle P1A_3_ play in the control of the myogenic tone has not yet been fully determined.

The activation of P1A_2A/B_ receptors is generally thought to mediate vasodilation in the coronary circulation in an endothelium-dependent and -independent manner via activation of the G_s_/AC/cAMP/PKA signaling pathway (86). In the coronary circulation, P1A_2A_-mediated vasorelaxation is achieved via modulation of both endothelial and smooth muscle cells (9, 86) and involves ATP-sensitive K^+^ (K_ATP_) channels (127). Up_4_A has been shown to mediate coronary smooth muscle relaxation through P1A_2A_-induced H_2_O_2_ production and subsequent activation of BK_Ca_ and voltage-gated K^+^ (K_V_) channels leading to vasodilation (87). A contractile role of P1A_2B_ in chorionic vascular smooth muscle cells has also been demonstrated (88). Here, activation of the P1A_2B_ is coupled to the synthesis of an arachidonate metabolite, likely thromboxane A_2_, which might activate a thromboxane receptor as the final effector of the adenosine contractile response.

## Vascular Smooth Muscle Purinergic Signaling in Hyperglycemia and Diabetes

The vascular system is severely impaired in response to hyperglycemia, affecting both endothelial, and vascular smooth muscle cells. Mechanisms mediating endothelial dysfunction during hyperglycemia have been extensively examined (19, 25, 28, 128–130), and therefore will not be further considered here. In diabetes, the distribution and location of purinergic receptors is altered and can also be shifted between endothelial and smooth muscle cells, which can affect vascular reactivity (69). In addition, hyperglycemia can alter vascular smooth muscle function and vascular reactivity, at least in part, through engagement of purinergic signaling (33, 49, 131). Indeed, elevations in extracellular glucose have been shown to induce a paracrine and/or autocrine release of nucleotides that could engage purinergic receptors to modulate vascular function (48, 49, 52). Purinergic receptors, but also nucleotide and nucleoside converting enzymes and transporters, are affected in the hyperglycemic vascular system (33). Surprisingly, the role of purinergic receptors in modulating vascular smooth muscle excitability in response to hyperglycemia and during diabetes has not been extensively examined. This open new opportunities for future research on how changes in the functional expression of purinergic receptors in vascular smooth muscle could impact myogenic tone and vascular reactivity during hyperglycemic and diabetic states.

The sympathetic system, through the release of ATP, is responsible for the activation of P2X_1_ receptors to induce vasoconstriction. Interestingly, accumulating data from animal and human studies suggest an overactivity of the sympathetic system as a defining factor in the development and maintenance of diabetes (132–134). In a type 2 animal model of diabetes [Goto-Kakizaki (GK) rats], Up4A-induced contraction of renal artery rings was shown to be increased due to the activation of the cyclooxygenase (COX)/thromboxane (Tx) receptor pathway (61). Similar observations were found in type 2 diabetic Otsuka Long-Evans Tokushima Fatty (OLETF) rats (135). In these OLETF rats, enhanced contraction was further increased with aging and suppressed by COX inhibition (135). Interestingly, increased Up4A concentration was detected in circulating plasma levels of human juvenile hypertensives (136). Furthermore, in mesenteric resistance arteries of diet-induced obesity rats, sympathetic nerve-mediated vasoconstriction is augmented, and involves upregulation of purinergic P2X_1_ signaling (62). Taken together, these studies suggest a potential role for P2X_1_ in vascular complications during hyperglycemia, obesity, and diabetes.

The role of P2Y_1_ in modulating vascular smooth muscle function in response to hyperglycemia and diabetes is unclear. Interestingly, however, a study employing P2Y_1_ knockout (${\text{P}2\text{Y}}_{1}^{-/-}$) mice, which present increased blood glucose levels (10 mM compared to 8 mM in wild type animals), showed that this receptor plays a physiological role in the maintenance of glucose homeostasis by regulating insulin secretion (137, 138). Although this mechanism is not directly involved in smooth muscle cell regulation of contractility, it highlights the need of subtype-specific purinergic receptor modulators for therapeutic use in order to avoid side-effects.

In mesenteric arteries from GK rats, the expression of P2Y_4_ was found to be decreased compared with control groups, and P2Y_2/4_-mediated contractions were shown to be increased (74). The diabetes-related enhancement of ATP-mediated vasoconstriction was due to P2Y receptor-dependent activation of the cPLA(2)/COX pathway. However, the cellular type (endothelial cell vs. smooth muscle cell) responsible for these effects was not identified in this study.

A role for P2Y_6_ receptors in modulating Ca^2+^ signaling and activation of the transcription factor NFATc3 during chronic elevations in extracellular glucose has been described (48). NFAT has been linked to vascular development during embryogenesis (139) and to cause enhanced vascular excitability in hypertension (140). Nilsson et al., demonstrated that high glucose promoted both nuclear translocation of NFATc3 and decreased its export from the nucleus via a P2Y_6_-dependent increase in intracellular Ca^2+^concentration (48). In this study, elevations in glucose (11.5–20 mmol/L) led to a global rise in intracellular calcium concentration through an autocrine/paracrine activation of P2Y_6_ receptors and subsequent activation of calcineurin, combined with inhibition of glycogen synthase kinase 3 (GSK3-b), and c-Jun N-terminal kinase (JNK). This combined action leads to an increase in NFAT nuclear accumulation and transcriptional activity, presenting this transcription factor as a P2Y_6_-dependent metabolic sensor in the vascular wall with relevance for vascular dysfunction in diabetes. Interestingly, NFAT3c activation has been shown to downregulate Kv channels (140) and BK_Ca_ channels (141), which indirectly increases L-type Ca^2+^ channel function leading to enhanced excitability of smooth muscle cells. Therefore, the activation of P2Y_6_ receptors following exposure to elevated levels of glucose could contribute to the activation of a molecular cascade leading to enhanced vascular contractility in diabetes.

In aorta of a mouse model of streptozotocin (STZ)-induced diabetes, P1A_1_ receptor-mediated signaling is modified without changes in overall protein expression (85). This study shows that P1A_1_ receptor-mediated vasoconstriction was decreased, and P1A_2A_ receptor-mediated vasodilation impaired in diabetic mice compared to control mice. The differences were attributed to changes in receptor sensitivity. The authors showed no differences in vascular reactivity in mesenteric arteries of diabetic mice, revealing the differences in tissue specific signaling. In addition, adenosine and adenosine receptors have important non-vascular regulatory roles on glucose homeostasis and lipid metabolism and therefore in the development of diabetes that can contribute to vascular complications observed during this pathological condition (142).

Recently, new data examining how acute hyperglycemia alters vascular smooth muscle excitability have revealed an unexpected role for a G_s_-coupled purinergic receptor that fits the profile of P2Y_11_ (49). Activation of this purinergic receptor in response to hyperglycemic conditions has been shown to modulate intracellular Ca^2+^ signaling and vascular reactivity (49). Given the unforeseen discovery of this P2Y_11_ in mediating the glucose effects in vascular smooth muscle as well as the novel signaling pathway described, we provide an extended discussion on this subject. Indeed, this study has great significance as it completely dissects the molecular events that link high glucose with changes in vascular reactivity in *ex vivo* and *in vivo* experiments utilizing both animal and human tissue.

### P2Y_11_ and cAMP/AC Signaling in Hyperglycemia

P2Y_11_ receptors were first cloned from human samples and thereafter in other species (59, 78). In a recent study, immunoreactive bands of expected and similar molecular weight for P2Y_11_ were detected in side-by-side samples of human adipose artery and mouse mesenteric artery lysates (49). Because the ubiquitous expression P2Y_11_ in many cell types, including endothelial cells, expression of P2Y_11_ was confirmed in freshly isolated human adipose, and mouse mesenteric vascular smooth muscle cells using immunofluorescence imaging. Although the gene for P2Y_11_ has not been found within the expected region of the mouse genome, a number of recent rodent annotations has been made (e.g., XM_008766009.2 and XM_0130655917.2) and a growing number of functional studies based on pharmacological data suggest at least the presence of a P2Y_11_-like receptor in rodents (78). Nevertheless, while further studies should be undertaken to confirm the P2Y_11_-like receptor in rodent tissue, data suggested the presence of a P2Y_11_/P2Y_11_-like receptors in human and murine vascular smooth muscle, respectively.

**Figure 1:**
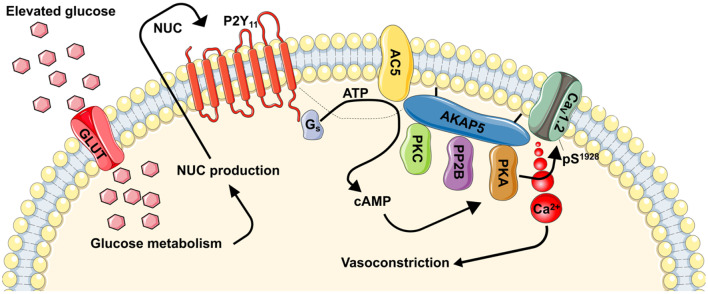
Proposed model for regulation of vascular contractility by P2Y_11_-dependent regulation of L-type Ca^2+^ channels during hyperglycemia and diabetes. During hyperglycemic conditions, glucose is transported into the cells via a glucose transporter (GLUT). Inside the cell, glucose is metabolized resulting in the production of nucleotides (NUC), such as ATP and UTP. These NUC are released to the extracellular space where they activate purinergic receptors coupled to G_s_ proteins (i.e., P2Y_11_). Activation of P2Y_11_ promotes AC5 activity and localized cAMP production. This cAMP microdomain can enable a pool of PKA that is intimately associated with L-type Ca^2+^ channels to increase Ca_V_1.2 phosphorylation at S1928, which will potentiate channel activity. Hyperactive L-type Ca^2+^ channels result in increased global [Ca^2+^]_i_ and contraction of vascular smooth muscle. Dotted line is to reflect potential close association between proteins. This figure was created using Servier Medical Art templates, which are licensed under a Creative Commons Attribution 3.0 Unported License; https://smart.servier.com.

Interest for a role for P2Y_11_ in glucose-induced changes in vascular smooth muscle Ca^2+^ homeostasis was fostered after the unexpected finding that elevated glucose could stimulate L-type Ca^2+^ channel activity leading to increased intracellular Ca^2+^ and vasoconstriction of mouse cerebral arteries via a mechanism requiring PKA (143, 144). P2Y_11_ is the only P2Y receptor subtype that couples to G_s_ proteins (145), which can stimulate AC activation to promote cAMP synthesis and PKA activity [Figure 1, (78, 146)]. Indeed, using innovative Förster resonance energy transfer (FRET) based cAMP biosensors expressed in human-derived tsA-201 cells, it was found that application of the highly selective P2Y_11_ agonist NF546 (147) increased cAMP synthesis (49). This NF546-induced cAMP response was blocked in cells treated with the selective P2Y_11_ inhibitor NF340 (147), but not with selective P2Y_1_ and P2Y_6_ inhibitors (49). In unpassaged human and mouse vascular smooth muscle cells expressing the same biosensor as above, similar NF546-induced cAMP responses were observed (49). Moreover, stimulating cells with an elevated glucose concentration (e.g., 15–20 mM D-glucose) that is comparable to that observed in diabetic patients and animal models (28, 143, 144, 148–150), cAMP synthesis of about the same magnitude as that observed with application of NF546 was observed. This response was not further amplified by the simultaneous application of both stimuli (49), suggesting that they may be acting via activation of the same signaling pathway. In addition, glucose-induced cAMP synthesis required glucose transport and metabolization as experiments in the presence of the membrane impermeable mannitol or non-metabolizable L-glucose failed to promote cAMP production (49). Importantly, the glucose and NF546 induced cAMP synthesis (as an independent stimulus or in combination) in human and mouse vascular smooth muscle cells was blocked if cells were first pre-treated with NF340. These findings provided robust data for the involvement of human P2Y_11_ and mouse P2Y_11_-like receptors in elevated glucose-induced cAMP synthesis.

The classic model for the production of cAMP by G_s_PCRs, including P2Y_11_/P2Y_11_-like receptors, suggest the involvement of intermediate regulatory enzymes such as AC [Figure 1, (151, 152)]. Nine membrane-bound AC isoforms have been described (153). Of these isoforms, AC3, AC5, and AC6 are the most abundantly expressed in vascular smooth muscle (154–156), and changes in their expression and/or function have been linked to vascular complications during diabetic hyperglycemia (157–159). AC3 and AC6 have been associated with vasodilatory pathways via regulation of K^+^ channels (155, 156). Moreover, a reduction in AC6 expression has been correlated with decreased vascular smooth muscle relaxation during diabetic hyperglycemia (158). Intriguingly a recent study revealed that AC5 was necessary for glucose-induced cAMP synthesis (160). Indeed, cAMP production in response to elevated glucose was completely prevented in vascular smooth muscle from AC5 knockout (AC5^−/−^), but not wild type or heterozygous (AC5^−/+^) mice. The lack of glucose-induced cAMP synthesis in AC5^−/−^ cells was comparable to results observed in wild type cells treated with the P2Y_11_ inhibitor NF340 (160). Thus, it is tempting to speculate that glucose-induced activation of P2Y_11_/P2Y_11_-like receptor leading to cAMP synthesis proceeds via engagement of AC5. This exciting possibility should be examined in future investigations.

### P2Y_11_, L-type Ca^2+^ Channel Ca_V_1.2, and Myogenic Tone in Hyperglycemia

Evidence linking hyperglycemia to changes in vascular smooth muscle intracellular Ca^2+^ dates back decades (161–163). Yet, the association between hyperglycemia, Ca^2+^ homeostasis and purinergic signaling was only reported about 13 years ago (48), and a more comprehensive mechanism is just surfacing [Figure 1, (49, 143, 144, 160, 164)]. In this emerging mechanism (and as stated above), glucose is transported into the cell likely via one or more glucose transporters. Indeed, vascular smooth muscle cells express insulin-independent (e.g., Glut1) and insulin-dependent (e.g., Glut4) glucose transporters (165–167). While there is evidence for a role for both pathways, pe-treatment with indinavir, which is consider a selective Glut4 inhibitor (168, 169), prevented glucose-mediated potentiation of L-type Ca^2+^ channels in isolated cerebral vascular smooth muscle cells (49). Once inside the cell, glucose is metabolized (49, 149). This promotes the release of nucleotides (48, 49, 52, 144, 170, 171), perhaps via pannexins, connexins, P2X_7_ receptors, or ATP-binding cassette transporters (13). These nucleotides activate P2Y_11_ receptors to stimulate the activity of coupled G_s_ proteins and subsequently AC to generate cAMP (Figure 1). Data suggest that the AC5 isoform underlies the glucose-induced cAMP synthesis (160), although more studies are needed to conclusively link P2Y_11_ with AC5. Glucose-induced production of cAMP then activates one of its effector proteins, in this case PKA, to stimulate L-type Ca^2+^ channel activity (Figure 1) (143, 144, 164). The increase in L-type Ca^2+^ channel activity is mediated by an elevation in the phosphorylation state of residue serine 1928 (S1928) in the pore-forming Ca_V_1.2 α1_c_ subunit (49, 144). This results in an increase in global intracellular Ca^2+^ in vascular smooth muscle cells to promote contraction and vasoconstriction (Figure 1). Glucose-induced vasoconstriction is observed in both in *ex vivo* and *in vivo* preparations (49, 144, 160), thus underscoring the significance of the activation of this signaling pathway.

Support for this mechanism is robust. First, glucose-induced L-type Ca^2+^ channel potentiation was prevented if glucose transport into vascular smooth muscle cells was blocked with the glucose transporter inhibitor indinavir (144). Second, the role of extracellular nucleotides was confirmed in experiments in which the ectonucleotidase apyrase prevented glucose-induced S1928 phosphorylation, L-type Ca^2+^ channel stimulation, and vasoconstriction (49). Moreover, experiments under continuous perfusion or static bath conditions corroborated the importance of extracellular nucleotides in potentiating L-type Ca^2+^ channel activity upon elevated glucose. Third, genetically depleting AC5 (e.g. AC5^−/−^) or pre-treating cells/tissue with the P2Y_11_ receptor inhibitor NF340 blocked glucose-induced L-type Ca^2+^ channel stimulation, global increases in intracellular Ca^2+^, and vasoconstriction (49, 160). Fourth, cell/arteries pre-treated with a PKA inhibitor (144) or from a S1928A mouse in which the phosphorylation of this amino acid residue is prevented (144, 172) failed to show S1928 phosphorylation, L-type Ca^2+^ channel potentiation, and vasoconstriction in response to elevated glucose or the P2Y_11_ agonist NF546. Based on these data, it is tempting to speculate that P2Y_11_, AC5, PKA, and Ca_V_1.2 are part of a signaling complex that facilitate its activation in response to elevations in extracellular glucose. Consistent with this possibility, super-resolution microscopy, and proximity ligation assay experiments have confirmed close association between subpopulations of these proteins, including Ca_V_1.2 and PKA (49, 144), Ca_V_1.2 and AC5 (160), and Ca_V_1.2 and P2Y_11_ (49). Further studies will be required to assess the interaction between all members of the signaling complex and not just their link to Ca_V_1.2.

A key question raised by the prior observations is what is the mechanism(s) for assembly of a potential P2Y_11_/AC5/PKA/Ca_V_1.2 signaling complex? It is well-known that compartmentalization of proteins is facilitated scaffold proteins such as AKAPs (151). The AKAP 5 isoform (AKAP5; murine AKAP150 and human AKAP79) is known to interact with AC5, PKA, and Ca_V_1.2 (173–178). Thus, AKAP5 could mediate AC5-mediated localized cAMP signaling and PKA compartmentalization to specifically stimulate vascular L-type Ca^2+^ channels upon elevated glucose. Evidence in support of this notion is provided by data indicating that genetic depletion of AKAP5 increases the distance between pools of Ca_V_1.2 and PKA and blocked S1928 phosphorylation, L-type Ca^2+^ channel potentiation, and vasoconstriction upon elevated glucose (144). Whether AKAP5 interacts with AC5 and P2Y_11_ in vascular smooth muscle, however, is unknown and should be investigated in future studies. These studies may confirm the formation of a unique signaling complex with broad implications in health and disease not only in vascular smooth muscle, but other excitable and non-excitable cells.

## Vascular Purinergic Signaling and Therapeutic

Drugs affecting purinoreceptor signaling have been tested in clinical trials for treating diabetic vascular complications. BVT-115959 (Cambridge Biotechnology), a selective P1A_2A_ agonist reached Phase II clinical trial for the treatment of neuropathic pain in diabetic patients with positive results (179). Unfortunately, the drug was discontinued as the company terminated its small molecule research program. Sonedenoson, a P1A_2A_ agonist, later found to be tissue plasminogen activator-dependent, entered a phase II clinical trial as a topical gel for the treatment of diabetic ulcers (180). Again, this trial was terminated due to poor enrollment. Polydeoxyribonucleotide (PDRN) has also been used for treating poorly vascularized foot ulcers by increasing neovascularization and angiogenesis and thought to be mediated through the activation of P1A_2A_ receptors (181). Although the effect of these drugs seems to be endothelium-dependent they present purinergic receptor modulation as a valid clinical strategy that could be extrapolated to vascular smooth muscle cells.

It is necessary to highlight current limitations for the use of purinergic modulators as therapeutic options and further research directions required. There is a lack of specific agonists/antagonists able to correctly distinguish between different purinergic subtypes. Investigations into basic mechanisms can overcome this issue by employing more generic modulators combined with the use of genetically manipulated systems or animal models, but in order to translate basic findings into therapeutic approaches there is an urgent need for better, more specific drugs. Furthermore, given the heterogeneity in tissue expression of purinergic receptors and the different functions carried in different cellular types (e.g., vascular smooth muscle cells vs. endothelial cells), basic research unraveling molecular cascades elicited by purinergic receptors will facilitate the development of more targeted and cell-specific therapeutic approaches.

## Conclusions

Purinergic signaling is a key modulator of vascular function and reactivity. In this review we have examined the expression, physiological role and their involvement in vascular physiology, and pathology. We focused on how purinergic receptors and downstream mediators are responsible for regulating vascular smooth muscle cell excitability. Furthermore, we have provided different examples in which this finely tuned purinergic system can be modified following hyperglycemia and diabetes. The pathophysiological roles of purinergic signaling in blood vessels are therefore clear. Given the availability of purinergic antagonists in clinical trials for different disorders, they represent promising therapeutic targets. However, it should be noted that given the ubiquity in expression of purinergic receptors, the selectivity of therapeutic strategies will be challenging. A major effort should be put into further understanding the interactions of purinergic signaling with other established signaling systems as well as in the development of inhibitors of extracellular ATP breakdown and transport in combination with more specific purinergic receptor agonists and antagonists.

Substantial efforts are being directed into understanding the mechanisms underlying enhanced vascular smooth muscle excitability during diabetes. In particular, the role of hyperglycemia in modifying the smooth muscle contractile state is the subject of intense investigation. Recent studies provided a direct link between high glucose and activation of P2Y_11_ leading to changes in vascular smooth muscle excitability via engagement of L-type Ca^2+^ channels (Figure 1). These studies have uncovered a detailed model of the molecular events that lead to altered vascular smooth muscle excitability and how this signaling response to elevations in extracellular glucose is compartmentalized. The clinical implications of this signaling complex are significant as they shed light on a mechanism underlying altered vascular reactivity during hyperglycemia and perhaps diabetes, providing novel targets that could be exploited for improving treatment of diabetic vasculopathy.

## Author Contributions

Each author wrote a section of the manuscript. M-MA integrated all the parts and generated figures and tables. MN provided overall supervision and direction to the project. All authors revised the manuscript and approved the final version.

## Conflict of Interest

The authors declare that the research was conducted in the absence of any commercial or financial relationships that could be construed as a potential conflict of interest.
